# Household income and health‐related quality of life in children receiving treatment for acute myeloid leukemia: Potential impact of selection bias in health equity research

**DOI:** 10.1002/cam4.6966

**Published:** 2024-04-04

**Authors:** Haley Newman, Yimei Li, Yuan‐Shung V. Huang, Caitlin W. Elgarten, Regina M. Myers, Jenny Ruiz, Daniel J. Zheng, Alison Barz Leahy, Catherine Aftandilian, Staci D. Arnold, Kira Bona, M. Monica Gramatges, Mallorie B. Heneghan, Kelly W. Maloney, Arunkumar J. Modi, Rajen J. Mody, Elaine Morgan, Jeffrey Rubnitz, Naomi Winick, Jennifer J. Wilkes, Alix E. Seif, Brian T. Fisher, Richard Aplenc, Kelly D. Getz

**Affiliations:** ^1^ Division of Oncology, Department of Pediatrics Children's Hospital of Philadelphia Philadelphia Pennsylvania USA; ^2^ Department of Pediatrics University of Pennsylvania Philadelphia Pennsylvania USA; ^3^ Department of Biostatistics, Epidemiology, and Informatics University of Pennsylvania Philadelphia Pennsylvania USA; ^4^ Department of Biomedical and Health Informatics Children's Hospital of Philadelphia Philadelphia Pennsylvania USA; ^5^ Division of Hematology‐Oncology, Department of Pediatrics UPMC Children's Hospital of Pittsburgh Pittsburgh Pennsylvania USA; ^6^ Division of Pediatric Hematology‐Oncology, Stem Cell Transplant and Regenerative Medicine, Department of Pediatrics Stanford University Stanford California USA; ^7^ Aflac Cancer and Blood Disorders Center, Children's Healthcare of Atlanta Emory University School of Medicine Atlanta Georgia USA; ^8^ Division of Population Sciences, Department of Pediatric Oncology Dana‐Farber Cancer Institute Boston Massachusetts USA; ^9^ Division of Pediatric Hematology‐Oncology, Department of Pediatrics Texas Children's Hospital, Baylor College of Medicine Houston Texas USA; ^10^ Division of Pediatric Hematology‐Oncology, Department of Pediatrics University of Utah Salt Lake City Utah USA; ^11^ Department of Pediatrics‐Hematology/Oncology and Bone Marrow Transplant, University of Colorado Cancer Center Children's Hospital Colorado Aurora Colorado USA; ^12^ Division of Hematology Oncology, Department of Pediatrics University of Arkansas for Medical Sciences, Arkansas Children's Hospital Little Rock Arkansas USA; ^13^ Department of Pediatrics University of Michigan Medical School Ann Arbor Michigan USA; ^14^ Department of Pediatrics Ann & Robert H. Lurie Children's Hospital of Chicago, Northwestern University Feinberg School of Medicine Chicago Illinois USA; ^15^ Department of Oncology St. Jude Children's Research Hospital Memphis Tennessee USA; ^16^ Department of Pediatric Hematology Oncology University of Texas Southwestern Medical Center Dallas Texas USA; ^17^ Division of Cancer and Blood Disorders, Department of Pediatrics University of Washington School of Medicine Seattle Washington USA; ^18^ Center for Childhood Cancer Research Children's Hospital of Philadelphia Philadelphia Pennsylvania USA; ^19^ Division of Infectious Disease, Department of Pediatrics Children's Hospital of Philadelphia Philadelphia Pennsylvania USA

**Keywords:** acute myeloid leukemia, epidemiology, pediatric cancer, quality of Life

## Abstract

**Objective:**

Examine the influence of household income on health‐related quality of life (HRQOL) among children with newly diagnosed acute myeloid leukemia (AML).

**Design:**

Secondary analysis of data prospectively collected from pediatric patients receiving treatment for AML at 14 hospitals across the United States.

**Exposure:**

Household income was self‐reported on a demographic survey. The examined mediators included the acuity of presentation and treatment toxicity.

**Outcome:**

Caregiver proxy reported assessment of patient HRQOL from the Peds QL 4.0 survey.

**Result:**

Children with AML (*n* = 131) and caregivers were prospectively enrolled to complete PedsQL assessments. HRQOL scores were better for patients in the lowest versus highest income category (mean ± SD: 76.0 ± 14 household income <$25,000 vs. 59.9 ± 17 income ≥$75,000; adjusted mean difference: 11.2, 95% CI: 2.2–20.2). Seven percent of enrolled patients presented with high acuity (ICU‐level care in the first 72 h), and 16% had high toxicity (any ICU‐level care); there were no identifiable differences by income, refuting mediating roles in the association between income and HRQOL. Enrolled patients were less likely to be Black/African American (9.9% vs. 22.2%), more likely to be privately insured (50.4% vs. 40.7%), and more likely to have been treated on a clinical trial (26.7% vs. 18.5%) compared to eligible unenrolled patients not enrolled. Evaluations of potential selection bias on the association between income and HRQOL suggested differences in HRQOL may be smaller than observed or even in the opposing direction.

**Conclusions:**

While primary analyses suggested lower household income was associated with superior HRQOL, differential participation may have biased these results. Future studies should partner with patients/families to identify strategies for equitable participation in clinical research.

## 1 INTRODUCTION

Pediatric acute myeloid leukemia (AML) is the second most common leukemia in children, with approximately 730 new cases in children and young adults under age 20 diagnosed in the US each year.^1^ Treatment is among the most intensive pediatric cancer protocols, with multiple chemotherapy cycles, prolonged hospital admissions, brief respite between cycles, and high rates of infectious complications. Although outcomes have improved in the past decades, approximately one‐third of patients experience relapse.^2^ Due to the treatment intensity and disease prognosis, among pediatric cancers, health‐related quality of life (HRQOL)—how illness impacts an individual's overall sense of wellbeing—may be uniquely impacted for children with AML.

Among children undergoing treatment for AML, individuals living in poverty may be especially vulnerable to impacts on HRQOL.^3^ Over the past decade, poverty has emerged as an important risk factor for inferior outcomes in pediatric cancer. Several studies have demonstrated that children living in poverty are more likely to relapse and die from their disease, even when treated on cooperative group clinical trials.^4^, ^5^, ^6^ Additionally, low‐income families report disproportionate income losses associated with cancer therapy,^7^ which may negatively impact quality of life.^8^, ^9^ Few studies have examined HRQOL in relation to household poverty in pediatric oncology,^3^, ^10^, ^11^, ^12^ and to our knowledge, none have explored this association in children undergoing AML treatment utilizing self‐reported household income rather than proxy measures of socioeconomic status. Additionally, few studies have evaluated potential mediators of the relationship between poverty exposure and HRQOL in cancer patients to guide meaningful interventions. Based on data suggesting that social determinants of health (SDOH)—including race and ethnicity and poverty exposure—are associated with delays in presentation, greater acuity at the time of diagnosis, and worse toxicity during therapy,^13^ we hypothesized that acuity of presentation and treatment toxicity as measured by ICU‐level care could potentially be mediators in the relationship between household income and HRQOL among children with newly diagnosed AML.

To address these knowledge gaps, we examined the association between caregiver‐reported household income and patient HRQOL during treatment for children and adolescents with newly diagnosed AML at 14 hospitals across the United States. We explored the potential role of presentation acuity and treatment toxicity as drivers of this association. We hypothesized that children from lower‐income households would have inferior HRQOL,^13^ and that this association would be mediated through higher rates of presentation acuity and toxicity during treatment.

## 2 METHODS

### 2.1 Source population

We performed a secondary analysis of data from an established multi‐institution cohort (Figure S1) of pediatric patients who received treatment for newly diagnosed AML between January 2011 and July 2019.^14^ Study protocols were approved by institutional review boards at all participating sites (Seattle Children's Hospital, Stanford Lucille Packard, Primary Children's Hospital, Colorado Children's Hospital, Dallas UT Southwestern, Houston Texas Children's Hospital, Arkansas Children's Hospital, Lurie Children's Hospital, CS Mott Children's Hospital, Detroit Children's Hospital, St Jude Research Hospital, Children's Hospital of Atlanta, Children's Hospital of Philadelphia, Dana‐Farber Cancer Institute). Patients were ineligible if they were older than 18 years at diagnosis, had a diagnosis of acute promyelocytic leukemia, or received only reduced‐intensity chemotherapy or hematopoietic stem cell transplant at the participating hospital.

### 2.2 Medical record abstraction

Trained medical record abstractors collected detailed demographic, clinical, diagnosis, treatment, and outcome data on all eligible patients. Institutional IRBs granted waivers of consent for electronic medical record (EMR) abstraction, allowing complete capture of the source population.

### 2.3 Recruitment and consent

The subset of the source population aged ≥2 years treated between June 2016 and May 2019 at 14 institutions and their caregivers were eligible to complete (1) a demographic survey including annual household income and (2) caregiver‐proxy reported assessments of patient HRQOL in English or Spanish. Site coordinators approached eligible patients/caregivers and documented assent/consent or reason for non‐participation.

Patients with incomplete household income/HRQOL data (*n* = 4) and patients with Trisomy 21 (*n* = 2) were excluded.

### 2.4 Exposure

Primary caregivers reported annual household income in the following categories: $<25,000; $25,000–$34,999; $35,000–49,999; $50,000–74,999; $75,000–99,999; $100,000–149,999; and $150,000+. Based on similarities in covariate distributions and mean HRQOL scores, income was collapsed into four categories ($<25,000; $25,000–49,999; $50,000–74,999; and $75,000+).

### 2.5 Outcome

Patient HRQOL was measured using caregiver‐proxy responses to the 23‐item acute PedsQL Generic Core Scales.^15^ Each question has a five‐point Likert response scale referencing the past 7 days: *never* (0), *almost never* (1), *sometimes* (2), *often* (3), *almost always* (4). Items are reverse scored and linearly transformed on a scale of 0–100, with a higher score indicating better HRQOL.^15^ Overall score as well as physical (physical functioning subscale) and psychosocial (emotional, social, and school functioning subscales) sub‐scores were evaluated.

### 2.6 Potential mediators

High presentation acuity and treatment toxicity were proposed as potential mediators of the relationship between income and patient HRQOL. High presentation acuity was defined as any ICU‐level resource utilization within the first 72 h following initial leukemia admission. High treatment toxicity was defined as any ICU‐level resource requirement from the start of systemic chemotherapy until the day prior to the HRQOL assessment.^13^, ^16^ The mediators were defined based on the occurrence of specific International Classification of Diseases Ninth and Tenth Revision (ICD‐9, ICD‐10) procedure codes or clinical resources considered a priori as markers of ICU‐level care^13^, ^17^ and were obtained via a merge with the Pediatric Health Information System database (PHIS). PHIS includes patient dates of service, diagnosis codes, procedure codes, and daily billing data for medications, laboratory tests, procedures, and other clinical resources.^16^, ^18^ Patients from institutions that did not contribute to PHIS were excluded from mediation analyses.

### 2.7 Covariates

Patient characteristics, including age, sex, race, ethnicity, risk classification, clinical trial enrollment, the chemotherapy cycle of HRQOL assessment, and primary insurance at diagnosis, were obtained from EMR abstraction. Given the small numbers of Black or African American patients (*n* = 13), independent associations could not be estimated; thus, race and ethnicity were examined as a combined variable (i.e., Hispanic, non‐Hispanic White, non‐Hispanic other, and ethnicity not reported). Exposure to inpatient versus outpatient neutropenia management during the treatment cycle preceding the HRQOL assessment was determined from abstracted chemotherapy administration, admission, and discharge data. Other characteristics, including caregiver education and employment, were obtained from the demographic survey.

### 2.8 Statistical analysis

Distributions of patient characteristics were compared by income categories using Fisher's exact test. Linear regressions compared PedsQL scores by income categories. Confounders were include in multivariable regressions if they were associated with the exposure (household income) with *p* < 0.2 or absolute difference in prevalence between income categories of ≥10% and outcome (HRQOL) with *p* < 0.2 or mean difference between income groups of ≥5%. For highly correlated covariates, the variable with the stronger association with income was included in the multivariable model.

To examine presentation acuity and treatment toxicity as potential mediators of the association between household income and HRQOL, the requisite component associations were assessed. The following were required for acuity or treatment toxicity to be considered potential mediators: statistically significant associations between (1) household income and acuity of presentation/treatment toxicity (modified Poisson regression), (2) acuity of presentation/treatment toxicity and HRQOL (linear regression), and (3) a non‐null total association between household income and HRQOL. Confounder selection mirrored primary analysis.

Evaluation of component associations were used to determine if mediation analyses were justified. If presentation acuity or treatment toxicity met the criteria of potential mediator, parameter estimates from the component associations would be combined to estimate the total association of income on HRQOL, its indirect component mediated through presentation acuity/treatment toxicity, and direct component through all other pathways.^19^ The proportion(s) of the total effect mediated through presentation acuity/treatment toxicity would be computed on the difference scale.^20^

### 2.9 Pre‐planned sensitivity analyses

To evaluate potential misclassification of exposure due to pre‐specified income categories, we compared self‐reported household income to primary insurance status at diagnosis (public only vs. private/other), considering public‐only insurance as a proxy for low household income. Patients with discordant income and insurance status (i.e., low‐income and private insurance; high‐income and public insurance, *n* = 8) were excluded in a restricted analysis. To assess the impact of differences in the chemotherapy cycle of HRQOL assessment, analysis restricted to patients surveyed during induction II was performed. To assess for potential over‐adjustment for structural racism, a multivariable analysis excluding race/ethnicity was also examined.^21^

### 2.10 Post hoc assessment for selection bias

Patient characteristics manually abstracted from the EMR were compared for patients who enrolled on the prospective study versus those who were eligible but did not enroll. Public‐only insurance at initial diagnosis was used as a proxy for low‐income patients who did not contribute demographic surveys and HRQOL assessments. To quantify the impact of selection bias on the primary results, analyses of the relationship between income and HRQOL were repeated, filling in missing HRQOL information for unenrolled patients under scenarios with varied assumptions.

One mechanism for selection bias is that low‐income patients were less likely to enroll, but enrollment was unrelated to HRQOL. In this scenario (scenario 1: least extreme), we assumed all unenrolled patients had PedsQL scores equal to the median score observed for enrolled patients, regardless of insurance status (income proxy). A second mechanism of selection bias is that low‐income patients with worse HRQOL were less likely to enroll, whereas enrollment for high‐income patients was unrelated to HRQOL. In this scenario (scenario 2: moderate), we assumed low‐income unenrolled patients had PedsQL scores equal to the 25th percentile observed for enrolled patients, and high‐income unenrolled patients had PedsQL scores equal to the median score observed for enrolled patients. A third possible scenario of selection bias was that low‐income patients with worse HRQOL were less likely to enroll, and high‐income patients with better HRQOL were also less likely to enroll (scenario 3: most extreme). In this scenario (scenario 3: extreme), we assumed that low‐income unenrolled patients had PedsQL scores equal to the observed 25th percentile, and high‐income patients had scores equal to the observed 75th percentile for enrolled patients. The assumption that lower‐income patients with worse HRQOL were less likely to enroll may be related to challenges with approach for study enrollment or possibly due to greater caregiver time constraints. The assumption that higher‐income families with better HRQOL were also less likely to enroll is potentially plausible if these families perceived a lack of relatability to study objectives.

### 2.11 Post hoc comparison of support structures

A survey of family support structures was added to prospective assessments part way through the prospective cohort study in response to themes identified in semi‐structured interviews with patients and caregivers.^22^, ^23^ Thus, information on support structures was available for a subset of families. As support structures may influence HRQOL, a post hoc assessment of support structures by income categories was performed (Table S1).

All analyses were performed using Stata (Stata, version 17.0; StataCorp).

## 3 RESULTS

### 3.1 Study population

**FIGURE 1:**
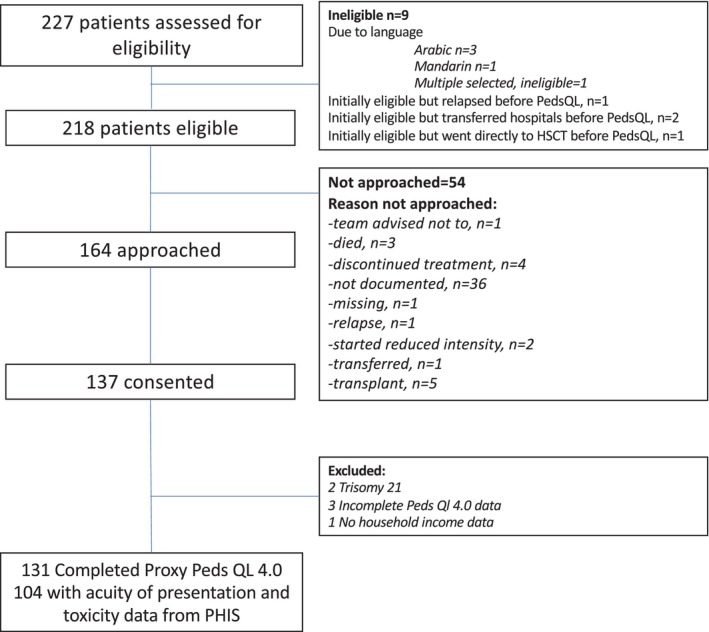
Flow diagram of patients eligible for enrollment, approached for study enrollment, and enrolled.

One hundred thirty‐one patients were included in the primary analysis of the association between income and HRQOL. Patients from PHIS‐contributing institutions (*n* = 104) were included in the analyses evaluating presentation acuity/treatment toxicity (Figure 1).

Patient characteristics by income are summarized in Table 1. Insurance status was collinear with income and thus not included in the multivariable adjustment. Age at diagnosis, sex, and race/ethnicity distributions varied by income. Patients in the lowest income categories were more likely to be Hispanic and had a greater frequency of caregiver unemployment compared to patients in the highest income category. There were modest differences in risk classification across income groups. The characteristics of the subpopulation of patients from PHIS‐contributing institutions were similar to the overall cohort (Table S2).

**TABLE 1:** Distribution of demographic and clinical characteristics by annual household income category.

	Total	<$25,000	$25,000–$49,999	$50,000–$74,999	≥$75,000	*p*
	*N* = 131	*N* = 28	*N* = 29	*N* = 18	*N* = 56	
*Demographics*						
Age at diagnosis						
0–4 years	52 (39.7%)	15 (53.6%)	8 (27.6%)	7 (38.9%)	22 (39.3%)	0.067
5–10 years	19 (14.5%)	6 (21.4%)	1 (3.4%)	3 (16.7%)	9 (16.1%)	
11–14 years	25 (19.1%)	4 (14.3%)	10 (34.5%)	4 (22.2%)	7 (12.5%)	
>15 years	35 (26.7%)	3 (10.7%)	10 (34.5%)	4 (22.2%)	18 (32.1%)	
Sex						
Female	56 (42.7%)	10 (35.7%)	18 (62.1%)	5 (27.8%)	23 (41.1%)	0.088
Male	75 (57.3%)	18 (64.3%)	11 (37.9%)	13 (72.2%)	33 (58.9%)	
Race/ethnicity						
Hispanic	21 (16.0%)	5 (17.9%)	11 (37.9%)	3 (16.7%)	2 (3.6%)	0.001
Non‐Hispanic White	69 (52.7%)	10 (35.7%)	11 (37.9%)	9 (50.0%)	39 (69.6%)	
Non‐Hispanic Other	26 (19.8%)	9 (32.1%)	6 (20.7%)	4 (22.2%)	7 (12.5%)	
Ethnicity not reported	15 (11.5%)	4 (14.3%)	1 (3.4%)	2 (11.1%)	8 (14.3%)	
Insurance status						
Private	66 (50.4%)	3 (10.7%)	6 (20.7%)	7 (38.9%)	50 (89.3%)	<0.001
Public	60 (45.8%)	23 (82.1%)	20 (69.0%)	11 (61.1%)	6 (10.7%)	
Missing	5 (3.8%)	2 (7.1%)	3 (10.3%)	0 (0.0%)	0 (0.0%)	
*Clinical characteristics*						
Risk classification						
Not high risk	53 (40.5%)	13 (46.4%)	16 (55.2%)	6 (33.3%)	18 (32.1%)	0.400
High risk	33 (25.2%)	7 (25.0%)	7 (24.1%)	4 (22.2%)	15 (26.8%)	
Missing	45 (34.4%)	8 (28.6%)	6 (20.7%)	8 (44.4%)	23 (41.1%)	
Inpatient or outpatient^a^						
Inpatient	43 (32.8%)	11 (39.3%)	10 (34.5%)	7 (38.9%)	15 (26.8%)	0.610
Outpatient	85 (64.9%)	17 (60.7%)	17 (58.6%)	11 (61.1%)	40 (71.4%)	
Missing	3 (2.3%)	0 (0.0%)	2 (6.9%)	0 (0.0%)	1 (1.8%)	
Clinical trial enrollment						
No	96 (73.3%)	22 (78.6%)	20 (69.0%)	13 (72.2%)	41 (73.2%)	0.880
Yes	35 (26.7%)	6 (21.4%)	9 (31.0%)	5 (27.8%)	15 (26.8%)	
Cycle of survey administration						
Induction II	92 (70.2%)	16 (57.1%)	21 (72.4%)	13 (72.2%)	42 (75.0%)	0.651
Intensification I	23 (17.6%)	7 (25.0%)	6 (20.7%)	3 (16.7%)	7 (12.5%)	
Intensification II	16 (12.2%)	5 (17.9%)	2 (6.9%)	2 (11.1%)	7 (12.5%)	
*Primary caregiver characteristics*						
Caregiver education level						
No college	37 (28.2%)	15 (53.6%)	16 (55.2%)	2 (11.1%)	4 (7.1%)	<0.001
Some college or greater	94 (71.8%)	13 (46.4%)	13 (44.8%)	16 (88.9%)	52 (92.9%)	
Caregiver employment						
Caregiver unemployed	54 (41.2%)	21 (75.0%)	13 (44.8%)	9 (50.0%)	11 (19.6%)	<0.001
Caregiver employed	76 (58.0%)	7 (25.0%)	15 (51.7%)	9 (50.0%)	45 (80.4%)	
Missing	1 (0.8%)	0 (0.0%)	1 (3.4%)	0 (0.0%)	0 (0.0%)	

*Note*: Values are expressed in *N* (%).

^a^ Outpatient is defined as discharged during neutropenia; prior literature demonstrates readmission rate during Induction II of 73%, with median time to readmission of 8 days (PMID 36795900).

### 3.2 Association between income and HRQOL

The overall mean PedsQL total score was 65.3 (range: 19.6, 94.4). There was a trend for a lower PedsQL total score (worse HRQOL) by increasing income category (Table 2; Table S3). Among the lowest income category (<$25,000), the mean PedsQL total score was 76.0 (SD 14.0) versus 59.9 (SD 17.4) in the highest income category (>$75,000). This pattern persisted following adjustments for age, sex, race/ethnicity, caregiver education, and employment. Psychosocial sub‐scores mirrored these associations. However, there were no statistically significant differences in physical health sub‐scores by income category. Physical sub‐scores were lowest across income categories. Results were consistent when restricting to the PHIS‐contributing subpopulation (Table S4).

**TABLE 2:** Peds QL 4.0 score^a^ by annual household income category.

Overall score			
Household income	Mean score (SD)	Adjusted mean difference (95% CI)^b^	*p*
<$25,000	76.0 (14.0)	11.2 (2.2–20.2)	0.015
$25,000–49,999	67.7 (18.4)	8.2 (−0.6 to 17.0)	0.069
$50,000–74,999	62.9 (19.4)	1.6 (−7.3 to 10.4)	0.727
≥$75,000	59.9 (17.4)	Ref	Ref

Psychosocial score			
Household income	Mean psychological health score (SD)	Adjusted mean difference (95% CI)^b^	*p*
<$25,000	78.0 (13.5)	13.8 (4.8–22.8)	0.003
$25,000–49,999	70.1 (16.9)	10.4 (1.6–19.2)	0.021
$50,000–74,999	66.3 (17.9)	3.3 (−5.5 to 19.2)	0.459
≥$75,000	62.4 (16.2)	Ref	Ref

Physical score			
Household income	Mean physical health score (SD)	Adjusted mean difference (95% CI)^b^	*p*
<$25,000	72.3 (18.9)	7.0 (−5.1 to 19.1)	0.253
$25,000–49,999	60.5 (27.3)	4.5 (−7.3 to 16.4)	0.450
$50,000–74,999	56.9 (25.9)	−0.9 (−12.9 to 11.0)	0.876
≥$75,000	54.7 (26.2)	Ref	Ref

^a^ Scores are on a scale of 0–100, with higher scores reflecting better caregiver‐proxy reported patient health‐related quality of life.

^b^ Model adjusted for age, sex, race/ethnicity, caregiver education, and caregiver employment status and has the highest income category as the reference group.

### 3.3 Assessment of mediators

Presentation acuity and treatment toxicity were explored as potential mediators of the relationship between income and HRQOL among patients with PHIS data. High presentation acuity was rare: only 6.7% of patients overall and one or two patients in each income category. The rarity of high presentation acuity precluded further evaluation as a potential mediator.

Approximately 16% of patients experienced high treatment toxicity, and the incidence of high treatment toxicity was not significantly different between income categories (Table 3). No clinical or demographic characteristics were associated with both income and treatment toxicity (Table S5); thus, adjustment was not warranted. Mean PedsQL scores were comparable for patients with high treatment toxicity (62.8, SD 4.4) and those without (66.6, SD 2.0). Given the absence of an association between income and high toxicity and high toxicity and HRQOL, toxicity did not meet the criteria of a mediator; thus, decomposition analyses were not performed.

**TABLE 3:** Presentation acuity and toxicity during treatment by annual household income category.^a^

Household income	High presentation acuity *N* = 7, 6.7%	*p*	Treatment Toxicity *N* = 17, 16.4%	*p*	RR of toxicity (95% CI)	*p*
<$25,000, *n* %	2, 9.5%	0.655	3, 14.3%	0.503	Ref	Ref
$25,000–$49,999, *n* %	1, 4.0%	–	3, 12%	–	0.8 (0.2–3.6)	0.779
$50,000–$74,999, *n* %	2, 11.8%	–	5, 29.4%	–	2.1 (0.6–7.4)	0.269
$75,000 and above, *n* %	2, 4.9%	–	6, 14.6%	–	1.0 (0.3–3.4)	0.943

^a^ 104 patients with acuity and toxicity data.

### 3.4 Pre‐planned sensitivity analyses

The association between income and HRQOL was similar when excluding patients with discordant income and insurance status (*n* = 9) and when restricting to patients who contributed HRQOL in induction II (*n* = 92). Results were also unchanged when excluding race/ethnicity from the multivariable model (Table S6).

### 3.5 Post hoc assessment for selection bias

A total of 218 patients were eligible for enrollment, of which 63% consented to enroll (Figure 1). Enrolled patients were less likely to be Black or African American (9.9% vs. 22.2%), more likely to be privately insured (50.4% vs. 40.7%), and more likely to have been treated on a clinical trial (26.7% vs. 18.5%) than eligible patients who did not enroll (Table S7).

Bias assessments were performed to assess the impact of selection bias on primary results (Table 4). Of 218 eligible patients, six were not include in the primary analysis (incomplete income or HRQOL data or Trisomy 21), and eight had unavailable insurance data to proxy income; thus, 204 were included in the bias assessment. Contrary to the results of our primary analysis, the moderate and most extreme bias scenarios demonstrated *worse* HRQOL for the low‐income group (public insurance) as compared with the higher‐income group (private/other insurance). In the least extreme scenario, while the direction of association was consistent with primary results (i.e., low‐income group having better HRQOL), the magnitude of the difference in PedsQL total score was substantially attenuated from 16 points to 4 points.

**TABLE 4:** Assessment of potential impacts of selection bias mechanisms—comparing HRQOL for both patients enrolled and not enrolled using public‐only insurance at initial diagnosis as a proxy for low household income. All scenarios added 37 unenrolled patients with private/other insurance and 41 unenrolled patients with public insurance.

Scenario 1. Least extreme scenario: low‐income patients were less likely to enroll, but enrollment was unrelated to HRQOL Assumption: All unenrolled patients had scores equal to the median of enrolled patients (score = 66.3)			
	Mean (SD)	Mean (SD)	0.0361
	Private or other	Public only	
General Core scale total score	63.5 (1.4)	67.7 (1.4)	
Scenario 2. Moderate scenario: low‐income patients with worse HRQOL were less likely to enroll, whereas enrollment for high‐income patients was unrelated to HRQOL Assumption: low‐income unenrolled patients had scores equal to the 25th percentile observed for enrolled patients (score = 34.8); high‐income unenrolled patients had scores equal to the median score observed for enrolled patients (score = 66.3)			
	Mean (SD)	Mean (SD)	0.0009
	Private or other	Public Only	
General Core scale total score	63.5 (1.8)	54.9 (1.8)	
Scenario 3. Extreme scenario: low‐income patients with worse HRQOL were less likely to enroll, and high‐income patients with better HRQOL were also less likely to enroll Assumption: low‐income unenrolled patients had scores of the observed 25th percentile (score = 34.8), and high‐income patients had scores equal to the observed 75th percentile for enrolled patients (score = 85.7)			
	Mean (SD)	Mean (SD)	<0.001
Overall score	Private or other	Public only	
General Core scale total score	70.4 (2.0)	54.9 (2.0)	

Abbreviation: HRQOL, health‐related quality of life.

### 3.6 Post hoc comparison of support structures

Among the subset of caregivers who completed the support structure questionnaire (*n* = 99), patients from high‐income households reported more support structures in most domains as compared with patients from low‐income households (Table S4).

## 4 DISCUSSION

We examined the association between household income and HRQOL in children receiving treatment for newly diagnosed AML. Overall, we found that HRQOL was low in all income groups as compared with healthy children^15^ and children with non‐cancerous chronic illness.^24^ Furthermore, contrary to our hypothesis, lower household income was associated with superior HRQOL. It is possible that for children from low‐income households, hospitalization for cancer therapy provides safety and reliability of housing and food that would otherwise be absent, which could positively influence HRQOL. However, further examination of participation in the prospective HRQOL assessments revealed that selection bias may have influenced our findings, as enrolled patients were less likely to be Black or African American, more likely to be privately insured, and more likely to have been treated on a clinical trial as compared to patients who were eligible for the study but did not enroll. Furthermore, results of analyses correcting for potential selection bias under various plausible scenarios demonstrated either no meaningful difference in patient HRQOL by household income or worse HRQOL among low‐income households compared to children from higher‐income households. Presentation acuity and treatment toxicity were rare and did not differ meaningfully by household income, and differential support structures did not explain observed associations between household income and HRQOL, as high‐income households reported more support structures as compared with low‐income households.

Worse patient HRQOL for low‐income families has been reported for pediatric acute lymphoblastic leukemia and a heterogeneous cohort of pediatric cancers.^3^, ^12^ Among children with AML enrolled on the Children's Oncology Group trial AAML1031, parent‐proxy reported HRQOL did not differ meaningfully by insurance status. However, approximately 50% of pediatric patients with AML do not enroll on clinical trials,^25^ and only a subset of enrolled patients participated in the HRQOL assessments.^10^ Thus prior findings may also be susceptible to selection bias.

Differential enrollment observed in our study is consistent with widely reported disparities in enrollment on oncology clinical trials and in trial‐embedded surveys.^10^, ^26^, ^27^ In adult cancer trials, patients of lower socioeconomic status have substantially lower rates of trial enrollment.^28^, ^29^ Additionally, publicly insured adolescents and young adults with various cancers are less likely to be enrolled on clinical trials,^30^ as are Black or African American children and young adults with AML.^25^, ^31^ While disparities in clinical trial enrollment are well documented, the potential for differential participation to bias study results or limit generalizability is underappreciated. Facilitators and barriers to participation may differ for historically marginalized populations, including children living in poverty. To ameliorate these disparities, research to understand the mechanisms of differential approach, enrollment, and retention is critical.

Our study has several limitations in addition to selection bias. There is the potential for outcome misclassification given that patient HRQOL was caregiver reported, which may be discordant with patient self‐report.^32^ Additionally, most patients contributed assessments in the second chemotherapy cycle; therefore, our results may not be generalizable to the totality of frontline treatment for AML. Furthermore, our cohort included small numbers, with a total eligible population of 218 patients, of which 63% consented to enroll.

In conclusion, primary analyses suggested that lower household income was associated with superior HRQOL, with no evidence of mediation through high presentation acuity or treatment toxicity. While we cannot rule out that the results of our primary analyses are true, our sensitivity analyses suggest these findings were impacted by selection bias. Our results further highlight the critical need to examine the presence of selection bias and the potential impact on study results to prevent misinterpreting or overinterpreting findings, particularly in disparities research. Future efforts should include mixed‐methods approaches, partnering with families from historically marginalized populations, and clinical staff to better understand facilitators and barriers to approach and enrollment. Such studies would provide important context for the patient experience before and during therapy. Interventions such as culturally informed patient navigation to assist with recruitment and retention; financial remuneration for time spent in study participation; and hospital‐based iterative assessments of diversity, equity, and inclusion on clinical trials and observational studies are strategies warranting further research to improve quality of life for all children with cancer.

## FUNDING INFORMATION

5K12CA076931‐24 to HN, K01HL143153 and R01HL164925 to KDG, PCORI CER‐1409‐22827 to RA, and Mai and Harry West Endowed Chair to RA.

## AUTHOR CONTRIBUTIONS

**Haley Newman:** Conceptualization (lead); formal analysis (lead); methodology (lead); writing – original draft (lead); writing – review and editing (equal). **Yimei Li:** Conceptualization (supporting); formal analysis (supporting); methodology (supporting); writing – original draft (supporting); writing – review and editing (equal). **Yuan‐Shung V. Huang:** Writing – review and editing (equal). **Caitlin W. Elgarten:** Writing – review and editing (equal). **Regina M. Myers:** Writing – review and editing (equal). **Jenny Ruiz:** Writing – review and editing (equal). **Daniel J. Zheng:** Writing – review and editing (equal). **Alison Barz Leahy:** Writing – review and editing (equal). **Catherine Aftandilian:** Writing – review and editing (equal). **Staci D. Arnold:** Writing – review and editing (equal). **Kira Bona:** Writing – review and editing (equal). **M. Monica Gramatges:** Writing – review and editing (equal). **Mallorie Heneghan B:** Writing – review and editing (equal). **Kelly Maloney:** Writing – review and editing (equal). **Arunkumar Modi J:** Writing – review and editing (equal). **Rajen Mody:** Writing – review and editing (equal). **Elaine Morgan:** Writing – review and editing (equal). **Jeffrey Rubnitz:** Writing – review and editing (equal). **Naomi J. Winick:** Writing – review and editing (equal). **Jennifer J. Wilkes:** Writing – review and editing (equal). **Alix E. Seif:** Writing – review and editing (equal). **Brian T. Fisher:** Writing – review and editing (equal). **Richard Aplenc:** Conceptualization (equal); writing – review and editing (equal). **Kelly D. Getz:** Conceptualization (equal); methodology (equal); supervision (equal); writing – original draft (equal); writing – review and editing (equal).

## CONFLICT OF INTEREST STATEMENT

None.

## Supporting information


Data S1:

## Data Availability

Statistical code and anonymized dataset are available upon request from the corresponding author.
