# Supplementary material for: Household income and health‐related quality of life in children receiving treatment for acute myeloid leukemia: Potential impact of selection bias in health equity research
Source: Cancer Med. 2024 Apr 4;13(7):e6966. doi: 10.1002/cam4.6966 (PMC10993703; doi:10.1002/cam4.6966)
Supplement: Supplementary file 1 — Data S1: [file CAM4-13-e6966-s001.zip › PCORI HRQOL Supplemental Figure legend and Tables_CANCER MED_DEC2023.docx]

Supplemental Figure Legend

Supplemental Figure 1. Map of contributing institutions participating in this study: 1– Seattle Children’s Hospital, 2 – Stanford Lucille Packard, 3– Primary Children’s Hospital , 4 – Colorado Children’s Hospital, 5 – Dallas UT Southwestern, 6 – Houston Texas Children’s Hospital, 7 – Arkansas Children’s Hospital, 8 – Lurie Children’s Hospital, 9 – CS Mott Children’s Hospital, 10 – Detroit Children’s Hospital, 11 – St Jude Research Hospital, 12 – Children’s Hospital of Atlanta, 13 – Children’s Hospital of Philadelphia, 14 – Dana Farber Cancer Institute

| Supplemental Table 1. Summary of the number of caregiver-reported sources of support by household income | | | | | | |
| --- | --- | --- | --- | --- | --- | --- |
|  | Total | <25000 | $25000-$49999/yr | $50000-$74999/yr | $75000 and above/yr | *P* |
|  | N=99^a^ | N=23 | N=20 | N=13 | N=43 |  |
| Childcare support | N(%) | N(%) | N(%) | N(%) | N(%) | 0.001 |
| 0 | 4 ( 4.0%) | 4 (17.4%) | 0 ( 0.0%) | 0 ( 0.0%) | 0 ( 0.0%) |  |
| 1 or 2 | 54 (54.5%) | 15 (65.2%) | 14 (70.0%) | 8 (61.5%) | 17 (39.5%) |  |
| 3 or more | 41 (41.4%) | 4 (17.4%) | 6 (30.0%) | 5 (38.5%) | 26 (60.5%) |  |
| Emotional support |  |  |  |  |  | 0.004 |
| none | 5 ( 5.1%) | 4 (17.4%) | 1 ( 5.0%) | 0 ( 0.0%) | 0 ( 0.0%) |  |
| 1 or 2 | 39 (39.4%) | 12 (52.2%) | 7 (35.0%) | 8 (61.5%) | 12 (27.9%) |  |
| 3 or more | 55 (55.6%) | 7 (30.4%) | 12 (60.0%) | 5 (38.5%) | 31 (72.1%) |  |
| Financial support |  |  |  |  |  | 0.280 |
| none | 16 (16.2%) | 6 (26.1%) | 2 (10.0%) | 3 (23.1%) | 5 (11.6%) |  |
| 1 or 2 | 58 (58.6%) | 15 (65.2%) | 11 (55.0%) | 6 (46.2%) | 26 (60.5%) |  |
| 3 or more | 25 (25.3%) | 2 ( 8.7%) | 7 (35.0%) | 4 (30.8%) | 12 (27.9%) |  |
| Logistic support^b^ |  |  |  |  |  | 0.004 |
| 0 to <1 | 9 ( 9.1%) | 4 (17.4%) | 3 (15.0%) | 2 (15.4%) | 0 ( 0.0%) |  |
| 1 to <3 | 65 (65.7%) | 18 (78.3%) | 13 (65.0%) | 7 (53.8%) | 27 (62.8%) |  |
| 3 or more | 25 (25.3%) | 1 ( 4.3%) | 4 (20.0%) | 4 (30.8%) | 16 (37.2%) |  |

a 99 enrolled patients reported support structures at the time of PedsQL assessment. Sources of support included: spouse/partner, other family member, friends, people at work, medical team, or other. Distributions by income compared using Fisher's exact test.

b Average number of sources of supports reported for: transportation, meals, and errands

| Supplemental Table 2. Distribution of demographic and clinical characteristics in overall cohort and sub-population of patients from PHIS contributing institutions. | | |
| --- | --- | --- |
|  | Total | PHIS |
|  | N=131 | N=104 |
| ***Demographics*** |  |  |
| Age at diagnosis, n % |  |  |
| 0-4 y | 39.7% | 40.4% |
| 5-10 y | 14.5% | 14.4% |
| 11-14 y | 19.1% | 18.3% |
| > 15 y | 26.7% | 26.9% |
| Sex, n % |  |  |
| Female | 42.7% | 42.3% |
| Male | 57.3% | 57.7% |
| Race/ethnicity, n % |  |  |
| Hispanic | 16.0% | 20.2% |
| Non-Hispanic White | 52.7% | 51.0% |
| Non-Hispanic Other | 19.8% | 21.2% |
| Ethnicity not reported | 11.5% | 7.7% |
| Insurance Status, n % |  |  |
| Private | 50.4% | 47.1% |
| Public | 45.8% | 50.0% |
| Missing | 3.8% | 2.9% |
| ***Clinical characteristics*** |  |  |
| Risk classification |  |  |
| Not high risk | 40.5% | 45.2% |
| High risk | 25.2% | 26.0% |
| Missing | 34.4% | 28.8% |
| Inpatient or outpatient |  |  |
| Inpatient | 32.8% | 34.6% |
| Outpatient | 64.9% | 64.4% |
|  | 2.3% | 1.0% |
| Clinical trial enrollment |  |  |
| No | 72.3% | 82.7% |
| Yes | 26.7% | 17.3% |
| ***Household characteristics*** |  |  |
| Caregiver education level |  |  |
| No college | 28.2% | 28.8% |
| Some college or greater | 71.8% | 71.2% |
| Caregiver employment |  |  |
| Unemployed | 41.2% | 43.3% |
| Employed | 58.0% | 56.7% |
| Missing | 0.8% | 0.0% |

| Supplemental Table 3. Peds QL 4.0 score^a^ by annual household income category. | | | | | | | | | | | | | |
| --- | --- | --- | --- | --- | --- | --- | --- | --- | --- | --- | --- | --- | --- |
| **OVERALL SCORE** |  |  |  |  |  |  |  |  |  |  |  |  |  |
| Household Income | Mean score (SD) | Unadjusted difference | *P* | Adjusted mean difference - age | *P* | Adjusted mean difference – age, sex | *P* | Adjusted mean difference – age, sex, race/ethnicity | *P* | Adjusted mean difference – age, sex, race/ethnicity, caregiver education | *P* | Adjusted mean difference – fully adjusted (95% CI)^b^ | *P* |
| < $25,000 | 76.0 (14.0) | 16.1 (8.1-24.0) | <0.001 | 13.7 (6.3-21.1) | <0.001 | 13.5 (6.2-20.9) | <0.001 | 13.1 (5.4-20.8) | 0.001 | 12.0 (3.7-20.4) | 0.005 | 11.2(2.2 to 20.2) | 0.015 |
| $25,000-49,999 | 67.7 (18.4) | 7.8 (-0.05-15.6) | 0.052 | 10.6 (3.1-18.0) | 0.006 | 11.6 (4.2-19.1) | 0.002 | 10.0 (2.0-18.2) | 0.015 | 9.2 (0.60-17.7) | 0.036 | 8.2(-0.6 to17.0) | 0.069 |
| $50,000-74,999 | 62.9 (19.4) | 3.0 (-6.3-12.2) | 0.524 | 3.2 (-5.4-11.8) | 0.464 | 2.5 (-6.0-11.1) | 0.558 | 2.0 (-6.6-10.7) | 0.640 | 2.1 (-6.6-10.7) | 0.640 | 1.6(-7.3 to 10.4) | 0.727 |
| >$75,000 | 59.9 (17.4) | ref | ref |  |  |  |  |  |  |  |  | ref | ref |
| **PSYCHOSOCIAL SCORE** |  |  |  |  |  |  |  |  |  |  |  |  |  |
| Household Income | Mean Psychological Health score (SD) | Unadjusted difference | *P* | Adjusted for age | *P* | Adjusted for age, sex | *P* | Adjusted age, sex, race/ethnicity | *P* | Adjusted age, sex, race/ethnicity, caregiver education | *P* | Adjusted mean difference (95% CI) ^b^ | *P* |
| < $25,000 | 78.0 (13.5) | 15.6 (8.3-22.9) | <0.001 | 14.6 (7.3-22.0) | <0.001 | 14.5 (7.2-21.7) | <0.001 | 14.6 (6.9-22.2) | <0.001 | 14.2 (5.9-22.5) | 0.001 | 13.8(4.8 to 22.8) | 0.003 |
| $25,000-49,999 | 70.1 (16.9) | 9.0 (1.7-16.2) | 0.016 | 10.4 (3.1-17.7) | 0.006 | 11.4 (4.0-18.7) | 0.003 | 11.2 (3.2-19.3) | 0.007 | 10.9 (2.4-19.4) | 0.013 | 10.4(1.6 to19.2) | 0.021 |
| $50,000-74,999 | 66.3 (17.9) | 3.9 (-4.7-12.5) | 0.377 | 4.2 (-4.3-12.6) | 0.330 | 3.6 (-4.8-12.0) | 0.401 | 3.5 (-5.0-12.1) | 0.415 | 3.5 (-5.1-12.2) | 0.417 | 3.3(-5.5 to 19.2) | 0.459 |
| >$75,000 | 62.4 (16.2) | ref | ref | ref | ref | Ref | Ref | Ref | Ref | Ref | ref | ref | ref |
| **PHYSICAL SCORE** |  |  |  |  |  |  |  |  |  |  |  |  |  |
| Household Income | Mean Physical Health score (SD) | Unadjusted difference | *P* | Adjusted for age | *P* | Adjusted for age, sex | *P* | Adjusted age, sex, race/ethnicity | *P* | Adjusted age, sex, race/ethnicity, caregiver education | *P* | Adjusted mean difference (95% CI) ^b^ | *P* |
| < $25,000 | 72.3 (18.9) | 17.6 (6.2-29.1) | 0.003 | 12.7 (2.5-22.9) | 0.015 | 12.5 (2.4-22.6) | 0.016 | 11.0 (0.59-21.4) | 0.039 | 8.6 (-2.6-19.9) | 0.312 | 7.0(-5.1 to 19.1) | 0.253 |
| $25,000-49,999 | 60.5 (27.3) | 5.9 (-5.5 – 17.2) | 0.308 | 11.3 (1.1-21.4) | 0.030 | 12.6 (2.4-22.9) | 0.016 | 8.5 (-2.46-19.4) | 0.127 | 6.4 (-5.1-18.0) | 0.273 | 4.5(-7.3 to 16.4) | 0.450 |
| $50,000-74,999 | 56.9 (25.9) | 2.1 (-11.3-15.5) | 0.755 | 2.1 (-9.6-13.9) | 0.721 | 1.3 (-10.4-13.0) | 0.824 | -0.03 (-11.7-11.7) | 0.996 | -0.02 (-11.7-11.7) | 0.997 | -0.9(-12.9 to 11.0) | 0.876 |
| >$75,000 | 54.7 (26.2) | ref | ref | Ref | ref | ref | Ref | Ref | Ref | Ref | ref | ref | ref |

| Supplemental Table 4. Peds QL 4.0 score^a^ by annual household income category among patients in the PHIS database (n=104). | | | |
| --- | --- | --- | --- |
| OVERALL SCORE | | | |
| Household Income | Mean score (SD) | Adjusted mean difference (95% CI)^b^ | *P* |
| < $25,000 | 77.3 (14.2) | 12.5 (2.7-22.3) | 0.013 |
| $25,000-49,999 | 65.3 (18.5) | 8.0 (-1.02-17.0) | 0.082 |
| $50,000-74,999 | 62.6 (19.9) | -0.46 (-10.2-9.3) | 0.926 |
| >$75,000 | 62.0 (17.3) | ref | ref |

a Scores are on a scale of 0-100 with higher scores reflecting better caregiver proxy reported patient health-related quality of life

b Model adjusted for age, sex, caregiver employment status

| Supplemental Table 5. Distribution of high acuity of presentation and treatment toxicity by demographics, clinical characteristics and household characteristics. | | | | |
| --- | --- | --- | --- | --- |
|  | Acuity | *P* | Toxicity | *P* |
| Overall, n % | N=7 |  | N=17 |  |
| ***Demographics*** |  |  |  |  |
| Age at diagnosis, n % |  | 0.35 |  | 0.48 |
| 0-4 y | 5 (12%) |  | 8 (19%) |  |
| 5-10 y | 0 ( 0%) |  | 2 (13%) |  |
| 11-14 y | 0 ( 0%) |  | 1 ( 5%) |  |
| > 15 y | 2 ( 7%) |  | 6 (21%) |  |
| Sex, n % |  | 0.70 |  | 1.00 |
| Female | 2 ( 5%) |  | 7 (16%) |  |
| Male | 5 ( 8%) |  | 10 (17%) |  |
| Race/ethnicity, n % |  | 0.008 |  | 0.44 |
| Hispanic | 2 (10%) |  | 3 (14%) |  |
| Non-Hispanic White | 1 ( 2%) |  | 8 (15%) |  |
| Non-Hispanic Other | 1 ( 5%) |  | 3 (14%) |  |
| Ethnicity not reported | 3 (38%) |  | 3 (38%) |  |
| Insurance Status, n % |  | 1.00 |  | 1.00 |
| Private | 3 ( 6%) |  | 8 (16%) |  |
| Public | 4 ( 8%) |  | 9 (17%) |  |
| ***Clinical characteristics*** |  |  |  |  |
| Risk classification |  | 0.54 |  | 0.56 |
| Not high risk | 3 ( 6%) |  | 9 (19%) |  |
| High risk | 3 (11%) |  | 5 (19%) |  |
| Missing | 1 ( 3%) |  | 3 (10%) |  |
| Inpatient or outpatient |  | 0.29 |  | 0.002 |
| Inpatient | 4 (11%) |  | 12 (33%) |  |
| Outpatient | 3 ( 4%) |  | 5 ( 7%) |  |
| Clinical trial enrollment |  | 0.35 |  | 0.17 |
| No | 5 ( 6%) |  | 12 (14%) |  |
| Yes | 2 (11%) |  | 5 (28%) |  |
| ***Household characteristics*** |  |  |  |  |
| Caregiver education level |  | 1.00 |  | 0.38 |
| No college | 4 ( 9%) |  | 3 (10%) |  |
| Some college or greater | 3 ( 5%) |  | 14 (19%) |  |
| Caregiver employment |  | 0.46 |  | 0.79 |
| Unemployed | 2 ( 7%) |  | 8 (18%) |  |
| Employed | 5 ( 7%) |  | 9 (15%) |  |

| Supplemental Table 6. Sensitivity analyses of the association between household income and Peds QL 4.0 score to address potential biases. | | | |
| --- | --- | --- | --- |
| **OVERALL SCORE**  Excluding patients with discordant household income and insurance status  (excluding high-income with public insurance, or low income with private insurance)  **N=122** | | | |
| **Household Income** | **Mean score (SD)** | **Adjusted mean difference (95% CI)**^a^ | **Adjusted *P* value** |
| < $25,000 | 76.1 (3.4) | 9.6 (0.27 to 18.9) | 0.044 |
| $25,000-49,999 | 67.2 (3.2) | 4.8 (-3.7 to 13.7) | 0.276 |
| $50,000-74,999 | 62.9 (4.0) | 1.7 (-7.3 to 10.6) | 0.715 |
| >$75,000 | 60.4 (2.4) | ref | ref |
| **OVERALL SCORE** Including only patients assessed for HRQOL in induction II  **N=92** | | | |
| **Household Income** | **Mean Score (SD)** | **Adjusted mean difference (95% CI)** ^b^ | **Adjusted *P* value** |
| < $25,000, n=16 | 72.5 (4.3) | 8.0 (-4.5 to 20.5) | 0.204 |
| $25,000-49,999, n=21 | 71.1 (3.8) | 12.3 (2.3 to 22.3) | 0.016 |
| $50,000-74,999, n=13 | 62.5 (4.8) | 3.8 (-6.8 to 14.4) | 0.477 |
| >$75,000, n=42 | 58.9 (2.7) | ref | ref |
| **OVERALL SCORE**  Excluding race/ethnicity from the original multivariate model  **N=131** | | | |
| **Household Income** | **Mean Score (SD)** | **Adjusted mean difference (95% CI)**^c^ | **Adjusted *P* value** |
| < $25,000 | 76.0 (3.3) | 11.7 (2.9 to 20.5) | 0.010 |
| $25,000-49,999 | 67.7 (3.1) | 9.8 (1.5 to 18.2) | 0.022 |
| $50,000-74,999 | 62.9 (4.0) | 2.2 (-6.7 to 10.9) | 0.625 |
| >$75,000 | 59.9 (2.3) | ref | ref |

a Model adjusted for age, race/ethnicity, risk classification, caregiver education, caregiver employment status

b Model adjusted for age, caregiver education, caregiver employment

c Model adjusted for age, sex, caregiver education, caregiver employment status

| Supplemental Table 7. Comparison of demographic and clinical characteristics for patients enrolled on this study versus patients who were eligible but not enrolled^a^ | | | |
| --- | --- | --- | --- |
| ***Demographics*** | Unenrolled patients  N=81 | Enrolled patients  N=131 | *P* |
| Age at diagnosis | N(%) | N(%) | 0.052 |
| 0-4 y | 28 (34.6%) | 52 (39.7%) |  |
| 5-10 y | 21 (25.9%) | 19 (14.5%) |  |
| 11-14 y | 20 (24.7%) | 25 (19.1%) |  |
| > 15 y | 12 (14.8%) | 35 (26.7%) |  |
| Sex |  |  | 0.035 |
| Female | 47 (58.0%) | 56 (42.7%) |  |
| Male | 34 (42.0%) | 75 (57.3%) |  |
| Race |  |  | 0.044 |
| White | 44 (54.3%) | 87 (66.4%) |  |
| Black or African American | 18 (22.2%) | 13 ( 9.9%) |  |
| Other | 19 (23.5%) | 31 (23.7%) |  |
| Race/ethnicity |  |  | 0.005 |
| Non-Hispanic White | 36 (44.4%) | 69 (52.7%) |  |
| Hispanic | 17 (21.0%) | 21 (16.0%) |  |
| Non-Hispanic Other | 27 (33.3%) | 26 (19.8%) |  |
| Ethnicity not reported | 1 ( 1.2%) | 15 (11.5%) |  |
| Risk classification |  |  | 0.21 |
| Not high risk | 23 (28.4%) | 53 (40.5%) |  |
| High risk | 25 (30.9%) | 33 (25.2%) |  |
| Missing | 33 (40.7%) | 45 (34.4%) |  |
| Clinical trial enrollment |  |  | 0.19 |
| No | 66 (81.5%) | 96 (73.3%) |  |
| Yes | 15 (18.5%) | 35 (26.7%) |  |
| Insurance Status |  |  | 0.053 |
| Private | 33 (40.7%) | 66 (50.4%) |  |
| Public | 41 (50.6%) | 60 (45.8%) |  |
| Other | 4 ( 4.9%) | 0 ( 0.0%) |  |
| Missing | 3 ( 3.7%) | 5 ( 3.8%) |  |
| Language |  |  | 0.17 |
| English | 67 (82.7%) | 120 (91.6%) |  |
| Spanish | 10 (12.3%) | 7 ( 5.3%) |  |
| Multiple | 2 ( 2.5%) | 2 ( 1.5%) |  |
| Other | 1 ( 1.2%) | 2 ( 1.5%) |  |
| Unknown | 1 ( 1.2%) | 0 ( 0.0%) |  |

a Total of 212 patients included; of 218 eligible, 6 enrolled but were not included in analysis (T21 n=2, incomplete income or QOL data n=4)
