# Supplementary figures and images for: Household income and health‐related quality of life in children receiving treatment for acute myeloid leukemia: Potential impact of selection bias in health equity research
Source: Cancer Med. 2024 Apr 4;13(7):e6966. doi: 10.1002/cam4.6966 (PMC10993703; doi:10.1002/cam4.6966)

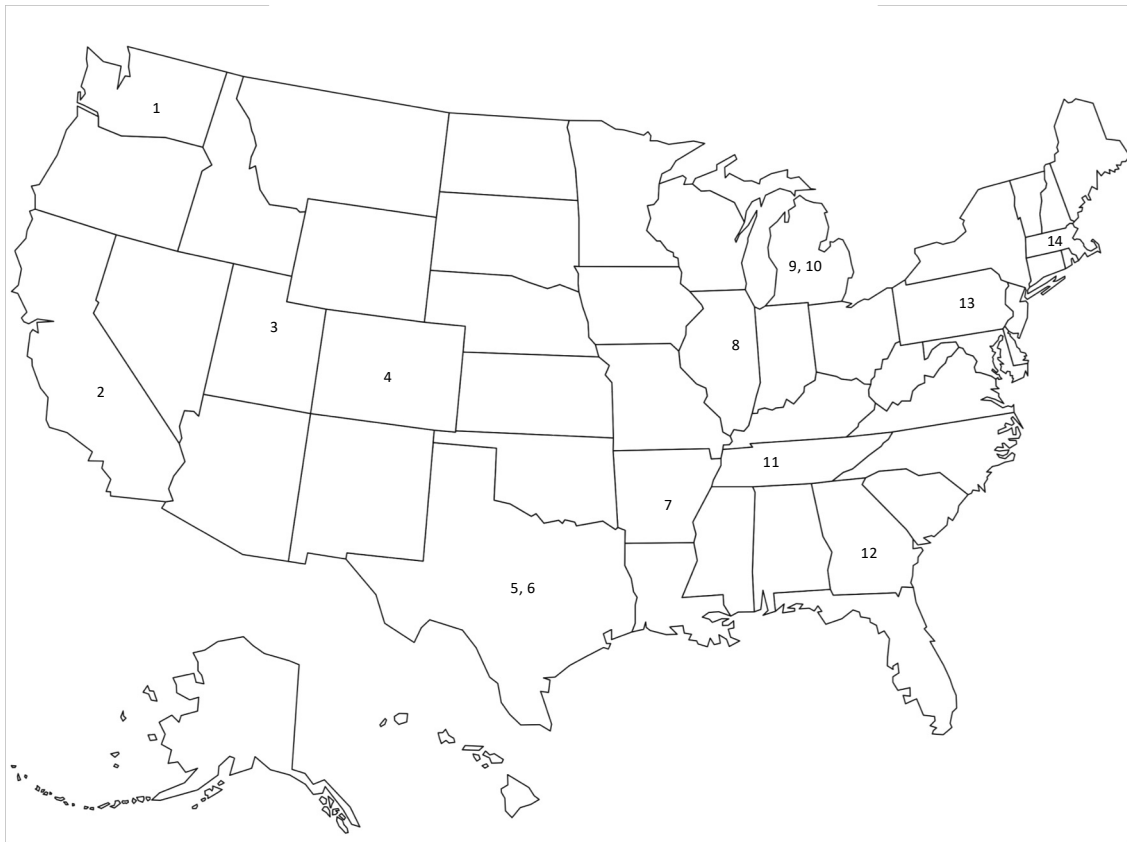

Supplement: Supplementary file 1 — Data S1: [file CAM4-13-e6966-s001.zip › Supplemental Figure 1.pdf]
